# Comparison of the crystal structures of 4,4′-bis­[3-(4-methyl­piperidin-1-yl)prop-1-yn-1-yl]-1,1′-biphenyl and 4,4′-bis­[3-(2,2,6,6-tetra­methyl­piperidin-1-yl)prop-1-yn-1-yl]-1,1′-biphen­yl

**DOI:** 10.1107/S2056989015015352

**Published:** 2015-09-12

**Authors:** Anqi Wan, Narsimha Reddy Penthala, E. Kim Fifer, Sean Parkin, Peter A. Crooks

**Affiliations:** a Department of Pharmaceutical Sciences, College of Pharmacy, University of Arkansas for Medical Sciences, Little Rock, AR 72205, USA; b Department of Chemistry, University of Kentucky, Lexington KY 40506, USA

**Keywords:** bis-tertiary ammonium analog, biphenyl ring, piperidine ring, crystal structure

## Abstract

As part of a comprehensive program to discover α9α10 nicotinic acetyl­choline receptor antagonists, the title compounds C_30_H_36_N_2_, (I), and C_36_H_48_N_2_, (II), were synthesized by coupling 4,4′-bis­(3-bromo­prop-1-yn-1-yl)-1,1′-biphenyl with 4-methyl­piperidine and 2,2,6,6-tetra­methyl­piperidine, respectively, in aceto­nitrile at room temperature. In compound (I), the biphenyl system has a twisted conformation with a dihedral angle of 26.57 (6)° between the two phenyl rings of the biphenyl moiety, while in compound (II), the biphenyl moiety sits on a crystallographic inversion centre so the two phenyl rings are exactly coplanar. The terminal piperidine rings in both compound (I) and compound (II) are in the chair conformation. In compound (I), the dihedral angles about the ethynyl groups between the planes of the phenyl rings and the piperidine ring N atoms are 37.16 (16) and 14.20 (17)°. In compound (II), the corresponding dihedral angles are both 61.48 (17)°. There are no noteworthy inter­molecular inter­actions in (I), but in (II) there is a small π-overlap between inversion-related mol­ecules (1 − *x*, 1 − *y*, 1 − *z*), with an inter­planar spacing of 3.553 (3) Å and centroid-to-centroid separation of 3.859 (4) Å.

## Abstract 2

The crystal structures of the two title compounds display chair conformations of the piperidine rings in their mol­ecules. In compound (I), the biphenyl system has a twisted conformation with a dihedral angle of 26.57 (6)° while in compound (II) the two phenyl rings are exactly coplanar.

## Chemical context   

Previous studies have shown that the bis-quaternary ammonium compound 1′-[(1,1′-biphen­yl)-4,4′-diylbis(prop-2-yne-3,1-di­yl)]bis­(3,4-di­methyl­pyridin-1-ium) bromide (ZZ161C) is a potent and selective α9α10 nicotinic acetyl­choline receptor antagonist (Zheng *et al.*, 2011 ▸). ZZ161C has been reported to have analgesic effects in various animal pain models (Wala *et al.*, 2012 ▸). In order to improve the pharmacological and pharmacokinetic profile of ZZ161C, we have replaced the terminal aza­aromatic rings with fully reduced piperidine rings to obtain the title compounds (I) and (II). Single-crystal X-ray structure determinations were carried out to determine the conformations of these compounds.

## Structural commentary   

**Figure 1:**
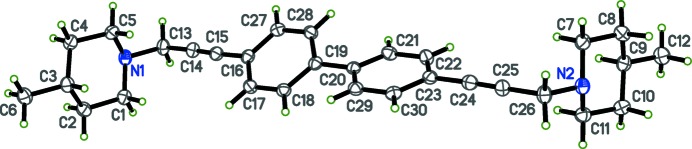
The mol­ecular structure of (I), with displacement ellipsoids drawn at the 50% probability level.

The title compounds, C_30_H_36_N_2_ (I) and C_36_H_48_N_2_ (II) are shown in Figs. 1 ▸ and 2 ▸, respectively. The present X-ray crystallographic study was carried out in order to ascertain the geometry of the piperidine rings and the biphenyl ring systems, as well as to obtain more detailed information about the conformation of the title compounds. Crystals of both (I) and (II) are monoclinic, space group *P*2_1_/*c*, with *Z*′ = 1 and 0.5, respectively. In each compound, individual bond lengths and angles are unremarkable. 
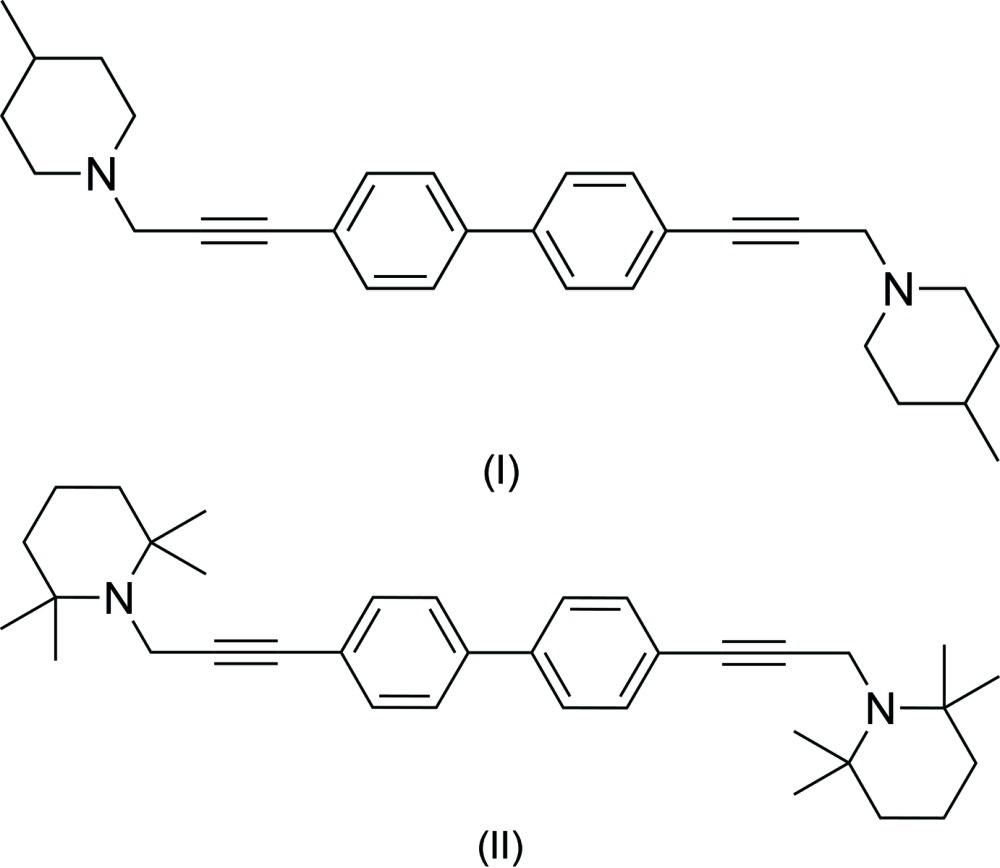


 The piperidine rings in both of the title mol­ecules are in the chair conformation. In (I), the biphenyl rings (C20-C21-C22-C23-C30-C29) and (C16-C17-C18-C19-C28-C27) are non-coplanar, with a dihedral angle of 26.57 (6)°. For compound (II), however, the biphenyl group is strictly coplanar because the mol­ecule sits on a crystallographic inversion centre. In compound (I), the dihedral angles about the ethynyl groups between the planes of the phenyl rings and the piperidine ring N atoms are 37.16 (16) and 14.20 (17)°. In compound (II), the corresponding dihedral angles are both 61.48 (17)°.

## Supra­molecular features   

Other than weak van der Waals inter­actions, there are no noteworthy inter­molecular contacts in (I). In (II) there is a small π-overlap between inversion-related mol­ecules (1 − *x*, 1 − *y*, 1 − *z*), giving an inter­planar spacing of 3.553 (3) Å and centroid-to-centroid separation of 3.859 (4) Å.

## Database survey   

A search of the November 2014 release of the Cambridge Structure Database (Groom & Allen, 2014 ▸), with updates through May 2015, using the program *Mogul* (Bruno *et al.*, 2004 ▸) for 4,4′ substituted biphenyl fragments was conducted. The search was restricted to non-organometallic, solvent-free structures with *R* < 5% and Cl as the heaviest element. There were over 1000 hits, which gave a bimodal distribution of biphenyl dihedral angles with a tight peak at 0° and a broader peak centred at 30°. The biphenyl dihedral angles in (I) and (II) are thus not unusual.

## Synthesis and crystallization   

In the synthesis of compound (I), 3,3′-[(1,1′-biphen­yl)-4,4′-di­yl]-bis­(prop-2-yn-1-ol) was synthesized by coupling 1,2,4,5-tetra­iodo­benzene with 4-pentyn-1-ol. Bis-(tri­phenyl­phos­phine)palladium(II) dichloride and copper(I) iodide were used as catalysts. The mixture was stirred at room temperature for 24 h under argon. The obtained 3,3′-[(1,1′-biphen­yl)-4,4′-di­yl]-bis­(prop-2-yn-1-ol) was converted to 4,4′-bis-(3-bromo­prop-1-yn-1-yl)-1,1′-biphenyl using bromo­methane and tri­phenyl­phosphine in anhydrous methyl­ene chloride at room temperature. To a suspension of the 4,4′-bis­(3-bromo­prop-1-yn-1-yl)-1,1′-biphenyl (100.0 mg, 0.26 mmol) in aceto­nitrile (7 mL) was added 4-methyl­piperidine (77.2 mg, 0.78 mmol) and the reaction mixture stirred for two hours at room temperature to obtain compound (I). Aceto­nitrile was removed from the reaction mixture under reduced pressure and the resulting residue was partitioned between water and di­chloro­methane. The organic layers were collected and combined. The extract (organic layer) was dried over anhydrous sodium sulfate, filtered, and the filtrate concentrated under reduced pressure. The resulting crude sample of compound (I) was purified by column chromatography (di­chloro­methane/methanol, 100:2 *v*/*v*). Yield: 80%.

A crude sample of compound (II) was prepared using the same experimental conditions for the preparation of compound (I) but utilizing 2,2,6,6-tetra­methyl­piperidine (110.0 mg, 0.78 mmol) instead of 4-methyl­piperidine. Column chromatography (dichlormethane/methanol 100:2 *v*/*v*) was then used for purification of (II). Yield: 80%.

Compound (I) and (II) were each dissolved separately in a mixture of di­chloro­methane/methanol (2:1 *v*/*v*). Yellow crystals of both compounds were obtained by slow evaporation of the solution at room temperature over 24 h.

Compound (I) ^1^H-NMR (400 Mz, CDCl_3_): δ 7.49 (*q*, 8H), 3.52 (*s*, 4H), 2.97 (*d*, 4H), 2.26 (*t*, 4H) p.p.m.; ^13^C-NMR (100 Mz, CDCl_3_): δ 132.92, 132.19, 126.76, 122.36, 85.13, 52.83, 48.09, 34.02, 30.20, 21.74 p.p.m.

Compound (II) ^1^H-NMR (400 Mz, CDCl_3_): δ 7.50 (*q*, 8H), 3.62 (*s*, 4H), 1.61–1.60 (*m*, 8H), 1.52–1.51 (*m*, 4H), 1.22 (*s*, 24H) p.p.m. ^13^C-NMR (100 Mz, CDCl_3_): δ 139.47, 131.88, 126.61, 123.42, 94.00, 80.78, 55.00, 41.16, 33.87, 27.49, 17.81 p.p.m.

## Refinement   

Crystal data, data collection and structure refinement details are summarized in Table 1 ▸. H atoms were found in difference Fourier maps, but subsequently included in the refinement using riding models, with constrained distances set to 0.95 Å (C*sp* ^2^H), 0.98 Å (*R*CH_3_), 0.99 Å (*R*_2_CH_2_) and 1.00 Å (*R*_3_CH). *U*_iso_(H) parameters were set to values of either 1.2*U*_eq_(C) or 1.5*U*_eq_(C) (*R*CH_3_ only) of the attached atom. The final models were checked using an *R*-tensor (Parkin, 2000 ▸) and by *PLATON* (Spek, 2009 ▸).

## Supplementary Material

Crystal structure: contains datablock(s) global, I, II. DOI: 10.1107/S2056989015015352/rz5164sup1.cif

Structure factors: contains datablock(s) I. DOI: 10.1107/S2056989015015352/rz5164Isup2.hkl

Structure factors: contains datablock(s) II. DOI: 10.1107/S2056989015015352/rz5164IIsup3.hkl

Supporting information file. DOI: 10.1107/S2056989015015352/rz5164Isup4.cml

Supporting information file. DOI: 10.1107/S2056989015015352/rz5164IIsup5.cml

CCDC references: 1419252, 1419251

Additional supporting information:  crystallographic information; 3D view; checkCIF report

## Figures and Tables

**Figure 2:**
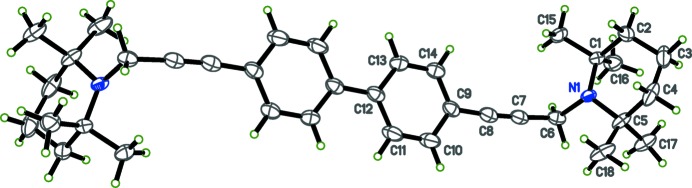
The mol­ecular structure of (II), with displacement ellipsoids drawn at the 50% probability level. Unlabelled atoms are generated by the symmetry operator (1 − *x*, 2 − *y*, 1 − *z*).

**Table 1:** Experimental details

	(I)	(II)
Crystal data		
Chemical formula	C_30_H_36_N_2_	C_36_H_48_N_2_
*M*_r_	424.61	508.76
Crystal system, space group	Monoclinic, *P*2_1_/*c*	Monoclinic, *P*2_1_/*c*
Temperature (K)	90	90
*a*, *b*, *c* (Å)	21.9870 (6), 7.0390 (3), 15.7840 (11)	16.0591 (3), 6.2267 (1), 15.5921 (3)
β (°)	99.0310 (19)	100.895 (1)
*V* (Å^3^)	2412.6 (2)	1531.03 (5)
*Z*	4	2
Radiation type	Mo *K*α	Cu *K*α
μ (mm^−1^)	0.07	0.47
Crystal size (mm)	0.32 × 0.30 × 0.03	0.22 × 0.04 × 0.03

Data collection		
Diffractometer	Nonius KappaCCD	Bruker X8 Proteum
Absorption correction	Multi-scan (*SADABS*; Sheldrick, 2008*a* ▸)	Multi-scan (*SADABS*; Bruker, 2006 ▸)
*T*_min_, *T*_max_	0.764, 0.958	0.767, 0.929
No. of measured, independent and observed [*I* > 2σ(*I*)] reflections	54455, 5546, 3347	19631, 2797, 2405
*R*_int_	0.066	0.053
(sin θ/λ)_max_ (Å^−1^)	0.650	0.602

Refinement		
*R*[*F* ^2^ > 2σ(*F* ^2^)], *wR*(*F* ^2^), *S*	0.049, 0.144, 1.02	0.050, 0.137, 1.05
No. of reflections	5546	2797
No. of parameters	291	176
H-atom treatment	H-atom parameters constrained	H-atom parameters constrained
Δρ_max_, Δρ_min_ (e Å^−3^)	0.22, −0.22	0.24, −0.24

Computer programs: *COLLECT* (Nonius, 1998 ▸), *APEX2* and *SAINT* (Bruker, 2006 ▸), *SCALEPACK* and *DENZO-SMN* (Otwinowski & Minor, 2006 ▸), *SHELXS97*, *SHELXTL* and *XP* in *SHELXTL* (Sheldrick, 2008*b* ▸), *SHELXL2014* (Sheldrick, 2015 ▸) and *CIFFIX* (Parkin, 2013 ▸).

## supplementary crystallographic information

### (I) 4,4'-Bis[3-(4-methylpiperidin-1-yl)prop-1-yn-1-yl]-1,1'-biphenyl Crystal data

**Table:**

C_30_H_36_N_2_	*F*(000) = 920
*M**_r_* = 424.61	*D*_x_ = 1.169 Mg m^−^^3^
Monoclinic, *P*2_1_/*c*	Mo *K*α radiation, λ = 0.71073 Å
*a* = 21.9870 (6) Å	Cell parameters from 5987 reflections
*b* = 7.0390 (3) Å	θ = 1.0–27.5°
*c* = 15.7840 (11) Å	µ = 0.07 mm^−^^1^
β = 99.0310 (19)°	*T* = 90 K
*V* = 2412.6 (2) Å^3^	Plate, colourless
*Z* = 4	0.32 × 0.30 × 0.03 mm

### (I) 4,4'-Bis[3-(4-methylpiperidin-1-yl)prop-1-yn-1-yl]-1,1'-biphenyl Data collection

**Table:**

Nonius KappaCCD diffractometer	5546 independent reflections
Radiation source: fine-focus sealed-tube	3347 reflections with *I* > 2σ(*I*)
Detector resolution: 9.1 pixels mm^-1^	*R*_int_ = 0.066
φ and ω scans at fixed χ = 55°	θ_max_ = 27.5°, θ_min_ = 1.9°
Absorption correction: multi-scan (*SADABS*; Sheldrick, 2008a)	*h* = −28→28
*T*_min_ = 0.764, *T*_max_ = 0.958	*k* = −9→9
54455 measured reflections	*l* = −20→20

### (I) 4,4'-Bis[3-(4-methylpiperidin-1-yl)prop-1-yn-1-yl]-1,1'-biphenyl Refinement

**Table:**

Refinement on *F*^2^	Primary atom site location: structure-invariant direct methods
Least-squares matrix: full	Secondary atom site location: difference Fourier map
*R*[*F*^2^ > 2σ(*F*^2^)] = 0.049	Hydrogen site location: difference Fourier map
*wR*(*F*^2^) = 0.144	H-atom parameters constrained
*S* = 1.02	*w* = 1/[σ^2^(*F*_o_^2^) + (0.0706*P*)^2^ + 0.3882*P*] where *P* = (*F*_o_^2^ + 2*F*_c_^2^)/3
5546 reflections	(Δ/σ)_max_ < 0.001
291 parameters	Δρ_max_ = 0.22 e Å^−^^3^
0 restraints	Δρ_min_ = −0.22 e Å^−^^3^

### (I) 4,4'-Bis[3-(4-methylpiperidin-1-yl)prop-1-yn-1-yl]-1,1'-biphenyl Special details

**Table:**

Experimental. The crystal was mounted with polyisobutene oil on the tip of a fine glass fibre, which was fastened in a copper mounting pin with electrical solder. It was placed directly into the cold gas stream of a liquid nitrogen based cryostat, according to published methods (Hope, 1994; Parkin & Hope, 1998).Diffraction data were collected with the crystal at 90 K, which is standard practice in this laboratory for the majority of flash-cooled crystals.
Geometry. All e.s.d.'s (except the e.s.d. in the dihedral angle between two l.s. planes) are estimated using the full covariance matrix. The cell e.s.d.'s are taken into account individually in the estimation of e.s.d.'s in distances, angles and torsion angles; correlations between e.s.d.'s in cell parameters are only used when they are defined by crystal symmetry. An approximate (isotropic) treatment of cell e.s.d.'s is used for estimating e.s.d.'s involving l.s. planes.
Refinement. Refinement progress was checked using *PLATON* (Spek, 2009) and by an *R*-tensor (Parkin, 2000). The final model was further checked with the IUCr utility *checkCIF*.

### (I) 4,4'-Bis[3-(4-methylpiperidin-1-yl)prop-1-yn-1-yl]-1,1'-biphenyl Fractional atomic coordinates and isotropic or equivalent isotropic displacement parameters (Å^2^)

**Table:**

	*x*	*y*	*z*	*U*_iso_*/*U*_eq_	
N1	0.20730 (5)	0.24205 (18)	0.41351 (8)	0.0245 (3)	
N2	0.79153 (5)	0.22850 (18)	−0.16448 (8)	0.0234 (3)	
C1	0.19401 (7)	0.4165 (2)	0.36333 (10)	0.0255 (4)	
H1A	0.2171	0.4153	0.3143	0.031*	
H1B	0.2080	0.5277	0.3996	0.031*	
C2	0.12538 (7)	0.4349 (2)	0.33012 (10)	0.0268 (4)	
H2A	0.1179	0.5510	0.2946	0.032*	
H2B	0.1027	0.4478	0.3793	0.032*	
C3	0.10128 (7)	0.2625 (2)	0.27674 (10)	0.0246 (4)	
H3A	0.1231	0.2569	0.2258	0.030*	
C4	0.11755 (6)	0.0832 (2)	0.32978 (10)	0.0259 (4)	
H4A	0.0947	0.0822	0.3791	0.031*	
H4B	0.1049	−0.0300	0.2941	0.031*	
C5	0.18653 (6)	0.0736 (2)	0.36255 (10)	0.0250 (4)	
H5A	0.1957	−0.0416	0.3982	0.030*	
H5B	0.2092	0.0644	0.3132	0.030*	
C6	0.03225 (7)	0.2769 (2)	0.24399 (10)	0.0304 (4)	
H6A	0.0188	0.1650	0.2091	0.046*	
H6B	0.0239	0.3918	0.2090	0.046*	
H6C	0.0098	0.2833	0.2929	0.046*	
C7	0.80978 (7)	0.0602 (2)	−0.11219 (10)	0.0251 (4)	
H7A	0.7993	−0.0555	−0.1470	0.030*	
H7B	0.7867	0.0566	−0.0631	0.030*	
C8	0.87867 (7)	0.0625 (2)	−0.07886 (10)	0.0263 (4)	
H8A	0.9017	0.0561	−0.1279	0.032*	
H8B	0.8897	−0.0505	−0.0424	0.032*	
C9	0.89727 (7)	0.2419 (2)	−0.02685 (10)	0.0248 (4)	
H9A	0.8756	0.2410	0.0243	0.030*	
C10	0.87516 (7)	0.4146 (2)	−0.08094 (10)	0.0264 (4)	
H10A	0.8981	0.4233	−0.1300	0.032*	
H10B	0.8838	0.5309	−0.0459	0.032*	
C11	0.80634 (7)	0.4027 (2)	−0.11454 (10)	0.0253 (4)	
H11A	0.7831	0.4048	−0.0657	0.030*	
H11B	0.7936	0.5146	−0.1511	0.030*	
C12	0.96641 (7)	0.2486 (2)	0.00546 (10)	0.0308 (4)	
H12A	0.9786	0.1362	0.0407	0.046*	
H12B	0.9763	0.3635	0.0400	0.046*	
H12C	0.9888	0.2505	−0.0436	0.046*	
C13	0.27358 (6)	0.2281 (2)	0.44724 (10)	0.0266 (4)	
H13A	0.2807	0.1132	0.4836	0.032*	
H13B	0.2858	0.3397	0.4842	0.032*	
C14	0.31350 (7)	0.2189 (2)	0.38025 (10)	0.0254 (4)	
C15	0.34323 (7)	0.2140 (2)	0.32287 (10)	0.0237 (3)	
C16	0.38357 (6)	0.2046 (2)	0.25885 (9)	0.0215 (3)	
C17	0.37212 (6)	0.3064 (2)	0.18216 (9)	0.0229 (3)	
H17A	0.3354	0.3788	0.1691	0.027*	
C18	0.41397 (6)	0.3028 (2)	0.12486 (9)	0.0214 (3)	
H18A	0.4054	0.3731	0.0730	0.026*	
C19	0.46856 (6)	0.1975 (2)	0.14209 (9)	0.0198 (3)	
C20	0.51543 (6)	0.2019 (2)	0.08399 (9)	0.0199 (3)	
C21	0.57773 (6)	0.1686 (2)	0.11555 (9)	0.0236 (4)	
H21A	0.5898	0.1423	0.1748	0.028*	
C22	0.62205 (7)	0.1732 (2)	0.06218 (9)	0.0250 (4)	
H22A	0.6640	0.1505	0.0852	0.030*	
C23	0.60560 (7)	0.2107 (2)	−0.02488 (10)	0.0225 (3)	
C24	0.64979 (7)	0.2140 (2)	−0.08346 (10)	0.0247 (4)	
C25	0.68349 (7)	0.2177 (2)	−0.13613 (10)	0.0244 (4)	
C26	0.72570 (6)	0.2217 (2)	−0.20057 (9)	0.0256 (4)	
H26A	0.7181	0.1073	−0.2372	0.031*	
H26B	0.7157	0.3341	−0.2379	0.031*	
C27	0.43683 (6)	0.0935 (2)	0.27488 (9)	0.0226 (3)	
H27A	0.4446	0.0193	0.3257	0.027*	
C28	0.47825 (6)	0.0904 (2)	0.21758 (9)	0.0223 (3)	
H28A	0.5141	0.0138	0.2297	0.027*	
C29	0.49939 (7)	0.2415 (2)	−0.00348 (10)	0.0214 (3)	
H29A	0.4576	0.2659	−0.0266	0.026*	
C30	0.54362 (7)	0.2455 (2)	−0.05680 (9)	0.0221 (3)	
H30A	0.5317	0.2723	−0.1161	0.027*	

### (I) 4,4'-Bis[3-(4-methylpiperidin-1-yl)prop-1-yn-1-yl]-1,1'-biphenyl Atomic displacement parameters (Å^2^)

**Table:**

	*U*^11^	*U*^22^	*U*^33^	*U*^12^	*U*^13^	*U*^23^
N1	0.0214 (6)	0.0309 (8)	0.0216 (7)	−0.0012 (5)	0.0049 (5)	−0.0001 (6)
N2	0.0216 (6)	0.0271 (8)	0.0218 (7)	−0.0004 (5)	0.0045 (5)	−0.0014 (5)
C1	0.0259 (8)	0.0268 (9)	0.0243 (8)	−0.0011 (7)	0.0061 (7)	−0.0036 (7)
C2	0.0278 (8)	0.0273 (9)	0.0255 (8)	0.0033 (7)	0.0053 (7)	−0.0013 (7)
C3	0.0228 (8)	0.0299 (9)	0.0216 (8)	0.0002 (6)	0.0051 (6)	−0.0005 (7)
C4	0.0244 (8)	0.0274 (9)	0.0257 (8)	−0.0029 (7)	0.0037 (7)	0.0010 (7)
C5	0.0256 (8)	0.0253 (9)	0.0247 (8)	−0.0018 (6)	0.0053 (7)	0.0016 (7)
C6	0.0267 (8)	0.0352 (10)	0.0284 (9)	0.0020 (7)	0.0014 (7)	0.0009 (7)
C7	0.0271 (8)	0.0243 (9)	0.0250 (8)	−0.0010 (7)	0.0071 (7)	−0.0026 (7)
C8	0.0263 (8)	0.0263 (9)	0.0263 (9)	0.0036 (7)	0.0044 (7)	−0.0008 (7)
C9	0.0262 (8)	0.0287 (9)	0.0202 (8)	−0.0008 (7)	0.0059 (6)	−0.0006 (7)
C10	0.0294 (8)	0.0242 (9)	0.0252 (8)	−0.0028 (7)	0.0028 (7)	0.0013 (7)
C11	0.0284 (8)	0.0234 (9)	0.0244 (8)	0.0008 (7)	0.0048 (7)	0.0014 (7)
C12	0.0283 (8)	0.0347 (10)	0.0285 (9)	0.0005 (7)	0.0024 (7)	−0.0014 (7)
C13	0.0210 (8)	0.0367 (10)	0.0226 (8)	−0.0013 (7)	0.0048 (6)	0.0010 (7)
C14	0.0227 (8)	0.0276 (9)	0.0257 (8)	−0.0022 (7)	0.0036 (7)	−0.0007 (7)
C15	0.0219 (8)	0.0221 (8)	0.0263 (8)	−0.0016 (6)	0.0014 (7)	−0.0010 (7)
C16	0.0219 (7)	0.0205 (8)	0.0222 (8)	−0.0036 (6)	0.0040 (6)	−0.0024 (6)
C17	0.0200 (7)	0.0214 (8)	0.0270 (8)	0.0011 (6)	0.0031 (6)	−0.0007 (7)
C18	0.0224 (7)	0.0207 (8)	0.0207 (8)	−0.0002 (6)	0.0019 (6)	0.0020 (6)
C19	0.0209 (7)	0.0180 (8)	0.0206 (8)	−0.0032 (6)	0.0033 (6)	−0.0027 (6)
C20	0.0219 (7)	0.0152 (8)	0.0232 (8)	−0.0004 (6)	0.0053 (6)	−0.0018 (6)
C21	0.0261 (8)	0.0231 (9)	0.0213 (8)	0.0035 (6)	0.0030 (7)	0.0015 (7)
C22	0.0210 (8)	0.0269 (9)	0.0271 (8)	0.0023 (6)	0.0041 (7)	0.0001 (7)
C23	0.0250 (8)	0.0181 (8)	0.0257 (8)	0.0013 (6)	0.0083 (7)	−0.0008 (7)
C24	0.0252 (8)	0.0225 (9)	0.0260 (8)	0.0009 (6)	0.0030 (7)	0.0004 (7)
C25	0.0221 (8)	0.0254 (9)	0.0259 (8)	−0.0001 (6)	0.0044 (7)	0.0000 (7)
C26	0.0231 (8)	0.0320 (9)	0.0224 (8)	−0.0001 (7)	0.0059 (6)	−0.0020 (7)
C27	0.0251 (8)	0.0213 (9)	0.0213 (8)	−0.0012 (6)	0.0034 (6)	0.0012 (6)
C28	0.0213 (7)	0.0205 (8)	0.0247 (8)	0.0018 (6)	0.0026 (6)	−0.0004 (6)
C29	0.0217 (7)	0.0178 (8)	0.0240 (8)	0.0001 (6)	0.0019 (6)	−0.0015 (6)
C30	0.0267 (8)	0.0197 (8)	0.0199 (8)	−0.0007 (6)	0.0034 (6)	0.0009 (6)

### (I) 4,4'-Bis[3-(4-methylpiperidin-1-yl)prop-1-yn-1-yl]-1,1'-biphenyl Geometric parameters (Å, º)

**Table:**

N1—C5	1.4647 (19)	C11—H11B	0.9900
N1—C1	1.4657 (19)	C12—H12A	0.9800
N1—C13	1.4741 (18)	C12—H12B	0.9800
N2—C7	1.4631 (19)	C12—H12C	0.9800
N2—C11	1.4666 (19)	C13—C14	1.478 (2)
N2—C26	1.4707 (18)	C13—H13A	0.9900
C1—C2	1.523 (2)	C13—H13B	0.9900
C1—H1A	0.9900	C14—C15	1.198 (2)
C1—H1B	0.9900	C15—C16	1.447 (2)
C2—C3	1.524 (2)	C16—C17	1.395 (2)
C2—H2A	0.9900	C16—C27	1.398 (2)
C2—H2B	0.9900	C17—C18	1.3880 (19)
C3—C4	1.526 (2)	C17—H17A	0.9500
C3—C6	1.5280 (19)	C18—C19	1.401 (2)
C3—H3A	1.0000	C18—H18A	0.9500
C4—C5	1.5252 (19)	C19—C28	1.3976 (19)
C4—H4A	0.9900	C19—C20	1.483 (2)
C4—H4B	0.9900	C20—C29	1.398 (2)
C5—H5A	0.9900	C20—C21	1.4019 (19)
C5—H5B	0.9900	C21—C22	1.3848 (19)
C6—H6A	0.9800	C21—H21A	0.9500
C6—H6B	0.9800	C22—C23	1.390 (2)
C6—H6C	0.9800	C22—H22A	0.9500
C7—C8	1.523 (2)	C23—C30	1.3984 (19)
C7—H7A	0.9900	C23—C24	1.443 (2)
C7—H7B	0.9900	C24—C25	1.197 (2)
C8—C9	1.527 (2)	C25—C26	1.481 (2)
C8—H8A	0.9900	C26—H26A	0.9900
C8—H8B	0.9900	C26—H26B	0.9900
C9—C10	1.520 (2)	C27—C28	1.3807 (19)
C9—C12	1.526 (2)	C27—H27A	0.9500
C9—H9A	1.0000	C28—H28A	0.9500
C10—C11	1.525 (2)	C29—C30	1.383 (2)
C10—H10A	0.9900	C29—H29A	0.9500
C10—H10B	0.9900	C30—H30A	0.9500
C11—H11A	0.9900		
			
C5—N1—C1	111.28 (11)	H10A—C10—H10B	108.0
C5—N1—C13	110.47 (12)	N2—C11—C10	110.91 (12)
C1—N1—C13	110.65 (12)	N2—C11—H11A	109.5
C7—N2—C11	110.83 (11)	C10—C11—H11A	109.5
C7—N2—C26	110.99 (12)	N2—C11—H11B	109.5
C11—N2—C26	110.93 (12)	C10—C11—H11B	109.5
N1—C1—C2	111.08 (12)	H11A—C11—H11B	108.0
N1—C1—H1A	109.4	C9—C12—H12A	109.5
C2—C1—H1A	109.4	C9—C12—H12B	109.5
N1—C1—H1B	109.4	H12A—C12—H12B	109.5
C2—C1—H1B	109.4	C9—C12—H12C	109.5
H1A—C1—H1B	108.0	H12A—C12—H12C	109.5
C1—C2—C3	111.25 (12)	H12B—C12—H12C	109.5
C1—C2—H2A	109.4	N1—C13—C14	114.15 (12)
C3—C2—H2A	109.4	N1—C13—H13A	108.7
C1—C2—H2B	109.4	C14—C13—H13A	108.7
C3—C2—H2B	109.4	N1—C13—H13B	108.7
H2A—C2—H2B	108.0	C14—C13—H13B	108.7
C2—C3—C4	108.91 (12)	H13A—C13—H13B	107.6
C2—C3—C6	112.03 (13)	C15—C14—C13	176.56 (16)
C4—C3—C6	112.04 (13)	C14—C15—C16	175.24 (15)
C2—C3—H3A	107.9	C17—C16—C27	118.45 (13)
C4—C3—H3A	107.9	C17—C16—C15	122.39 (13)
C6—C3—H3A	107.9	C27—C16—C15	119.14 (13)
C5—C4—C3	110.95 (12)	C18—C17—C16	120.54 (13)
C5—C4—H4A	109.4	C18—C17—H17A	119.7
C3—C4—H4A	109.4	C16—C17—H17A	119.7
C5—C4—H4B	109.4	C17—C18—C19	121.25 (14)
C3—C4—H4B	109.4	C17—C18—H18A	119.4
H4A—C4—H4B	108.0	C19—C18—H18A	119.4
N1—C5—C4	110.99 (12)	C28—C19—C18	117.53 (13)
N1—C5—H5A	109.4	C28—C19—C20	120.70 (13)
C4—C5—H5A	109.4	C18—C19—C20	121.75 (13)
N1—C5—H5B	109.4	C29—C20—C21	117.70 (13)
C4—C5—H5B	109.4	C29—C20—C19	121.51 (13)
H5A—C5—H5B	108.0	C21—C20—C19	120.79 (13)
C3—C6—H6A	109.5	C22—C21—C20	121.44 (14)
C3—C6—H6B	109.5	C22—C21—H21A	119.3
H6A—C6—H6B	109.5	C20—C21—H21A	119.3
C3—C6—H6C	109.5	C21—C22—C23	120.46 (14)
H6A—C6—H6C	109.5	C21—C22—H22A	119.8
H6B—C6—H6C	109.5	C23—C22—H22A	119.8
N2—C7—C8	110.85 (12)	C22—C23—C30	118.49 (13)
N2—C7—H7A	109.5	C22—C23—C24	122.66 (13)
C8—C7—H7A	109.5	C30—C23—C24	118.85 (14)
N2—C7—H7B	109.5	C25—C24—C23	175.97 (16)
C8—C7—H7B	109.5	C24—C25—C26	179.41 (16)
H7A—C7—H7B	108.1	N2—C26—C25	114.80 (12)
C7—C8—C9	111.25 (12)	N2—C26—H26A	108.6
C7—C8—H8A	109.4	C25—C26—H26A	108.6
C9—C8—H8A	109.4	N2—C26—H26B	108.6
C7—C8—H8B	109.4	C25—C26—H26B	108.6
C9—C8—H8B	109.4	H26A—C26—H26B	107.5
H8A—C8—H8B	108.0	C28—C27—C16	120.67 (14)
C10—C9—C12	112.14 (13)	C28—C27—H27A	119.7
C10—C9—C8	108.91 (12)	C16—C27—H27A	119.7
C12—C9—C8	111.96 (12)	C27—C28—C19	121.47 (14)
C10—C9—H9A	107.9	C27—C28—H28A	119.3
C12—C9—H9A	107.9	C19—C28—H28A	119.3
C8—C9—H9A	107.9	C30—C29—C20	120.84 (14)
C9—C10—C11	111.35 (12)	C30—C29—H29A	119.6
C9—C10—H10A	109.4	C20—C29—H29A	119.6
C11—C10—H10A	109.4	C29—C30—C23	121.06 (14)
C9—C10—H10B	109.4	C29—C30—H30A	119.5
C11—C10—H10B	109.4	C23—C30—H30A	119.5
			
C5—N1—C1—C2	58.26 (15)	C16—C17—C18—C19	0.1 (2)
C13—N1—C1—C2	−178.52 (12)	C17—C18—C19—C28	2.4 (2)
N1—C1—C2—C3	−56.80 (17)	C17—C18—C19—C20	−176.17 (13)
C1—C2—C3—C4	54.60 (16)	C28—C19—C20—C29	154.84 (14)
C1—C2—C3—C6	179.11 (12)	C18—C19—C20—C29	−26.7 (2)
C2—C3—C4—C5	−54.81 (16)	C28—C19—C20—C21	−26.0 (2)
C6—C3—C4—C5	−179.30 (12)	C18—C19—C20—C21	152.48 (14)
C1—N1—C5—C4	−58.59 (15)	C29—C20—C21—C22	−0.5 (2)
C13—N1—C5—C4	178.08 (12)	C19—C20—C21—C22	−179.67 (14)
C3—C4—C5—N1	57.36 (17)	C20—C21—C22—C23	−0.2 (2)
C11—N2—C7—C8	−59.31 (15)	C21—C22—C23—C30	0.7 (2)
C26—N2—C7—C8	176.95 (12)	C21—C22—C23—C24	−178.97 (14)
N2—C7—C8—C9	57.45 (17)	C7—N2—C26—C25	61.28 (17)
C7—C8—C9—C10	−54.19 (16)	C11—N2—C26—C25	−62.40 (17)
C7—C8—C9—C12	−178.78 (12)	C17—C16—C27—C28	2.4 (2)
C12—C9—C10—C11	178.51 (12)	C15—C16—C27—C28	−176.02 (13)
C8—C9—C10—C11	54.03 (16)	C16—C27—C28—C19	0.0 (2)
C7—N2—C11—C10	59.11 (15)	C18—C19—C28—C27	−2.4 (2)
C26—N2—C11—C10	−177.12 (12)	C20—C19—C28—C27	176.14 (13)
C9—C10—C11—N2	−57.14 (17)	C21—C20—C29—C30	0.7 (2)
C5—N1—C13—C14	61.90 (17)	C19—C20—C29—C30	179.85 (13)
C1—N1—C13—C14	−61.79 (17)	C20—C29—C30—C23	−0.2 (2)
C27—C16—C17—C18	−2.5 (2)	C22—C23—C30—C29	−0.5 (2)
C15—C16—C17—C18	175.94 (14)	C24—C23—C30—C29	179.17 (13)

### (II) 4,4'-Bis[3-(2,2,6,6-tetramethylpiperidin-1-yl)prop-1-yn-1-yl]-1,1'-biphenyl Crystal data

**Table:**

C_36_H_48_N_2_	*F*(000) = 556
*M**_r_* = 508.76	*D*_x_ = 1.104 Mg m^−^^3^
Monoclinic, *P*2_1_/*c*	Cu *K*α radiation, λ = 1.54178 Å
*a* = 16.0591 (3) Å	Cell parameters from 8231 reflections
*b* = 6.2267 (1) Å	θ = 2.8–67.9°
*c* = 15.5921 (3) Å	µ = 0.47 mm^−^^1^
β = 100.895 (1)°	*T* = 90 K
*V* = 1531.03 (5) Å^3^	Needle, colourless
*Z* = 2	0.22 × 0.04 × 0.03 mm

### (II) 4,4'-Bis[3-(2,2,6,6-tetramethylpiperidin-1-yl)prop-1-yn-1-yl]-1,1'-biphenyl Data collection

**Table:**

Bruker X8 Proteum diffractometer	2797 independent reflections
Radiation source: fine-focus rotating anode	2405 reflections with *I* > 2σ(*I*)
Detector resolution: 5.6 pixels mm^-1^	*R*_int_ = 0.053
φ and ω scans	θ_max_ = 68.2°, θ_min_ = 2.8°
Absorption correction: multi-scan (*SADABS*; Bruker, 2006)	*h* = −19→18
*T*_min_ = 0.767, *T*_max_ = 0.929	*k* = −7→7
19631 measured reflections	*l* = −7→18

### (II) 4,4'-Bis[3-(2,2,6,6-tetramethylpiperidin-1-yl)prop-1-yn-1-yl]-1,1'-biphenyl Refinement

**Table:**

Refinement on *F*^2^	Primary atom site location: structure-invariant direct methods
Least-squares matrix: full	Secondary atom site location: difference Fourier map
*R*[*F*^2^ > 2σ(*F*^2^)] = 0.050	Hydrogen site location: difference Fourier map
*wR*(*F*^2^) = 0.137	H-atom parameters constrained
*S* = 1.05	*w* = 1/[σ^2^(*F*_o_^2^) + (0.0655*P*)^2^ + 0.6795*P*] where *P* = (*F*_o_^2^ + 2*F*_c_^2^)/3
2797 reflections	(Δ/σ)_max_ < 0.001
176 parameters	Δρ_max_ = 0.24 e Å^−^^3^
0 restraints	Δρ_min_ = −0.24 e Å^−^^3^

### (II) 4,4'-Bis[3-(2,2,6,6-tetramethylpiperidin-1-yl)prop-1-yn-1-yl]-1,1'-biphenyl Special details

**Table:**

Geometry. All e.s.d.'s (except the e.s.d. in the dihedral angle between two l.s. planes) are estimated using the full covariance matrix. The cell e.s.d.'s are taken into account individually in the estimation of e.s.d.'s in distances, angles and torsion angles; correlations between e.s.d.'s in cell parameters are only used when they are defined by crystal symmetry. An approximate (isotropic) treatment of cell e.s.d.'s is used for estimating e.s.d.'s involving l.s. planes.
Refinement. Refinement progress was checked using *PLATON* (Spek, 2009) and by an *R*-tensor (Parkin, 2000). The final model was further checked with the IUCr utility *checkCIF*.

### (II) 4,4'-Bis[3-(2,2,6,6-tetramethylpiperidin-1-yl)prop-1-yn-1-yl]-1,1'-biphenyl Fractional atomic coordinates and isotropic or equivalent isotropic displacement parameters (Å^2^)

**Table:**

	*x*	*y*	*z*	*U*_iso_*/*U*_eq_	
N1	0.78341 (8)	0.0593 (2)	0.79190 (7)	0.0251 (3)	
C1	0.83571 (10)	−0.0569 (3)	0.73835 (10)	0.0282 (4)	
C2	0.92701 (10)	0.0213 (3)	0.76309 (11)	0.0362 (4)	
H2A	0.9634	−0.0696	0.7333	0.043*	
H2B	0.9303	0.1703	0.7418	0.043*	
C3	0.96116 (11)	0.0163 (3)	0.86058 (12)	0.0459 (5)	
H3A	1.0197	0.0734	0.8732	0.055*	
H3B	0.9623	−0.1334	0.8822	0.055*	
C4	0.90420 (12)	0.1523 (3)	0.90597 (11)	0.0450 (5)	
H4A	0.9072	0.3034	0.8869	0.054*	
H4B	0.9258	0.1472	0.9698	0.054*	
C5	0.81150 (11)	0.0802 (3)	0.88750 (9)	0.0344 (4)	
C6	0.69196 (10)	0.0225 (3)	0.76398 (11)	0.0311 (4)	
H6A	0.6669	−0.0110	0.8158	0.037*	
H6B	0.6829	−0.1031	0.7244	0.037*	
C7	0.64850 (9)	0.2102 (3)	0.71880 (10)	0.0315 (4)	
C8	0.61556 (10)	0.3635 (3)	0.68003 (10)	0.0321 (4)	
C9	0.58105 (9)	0.5448 (3)	0.62925 (10)	0.0306 (4)	
C10	0.52059 (11)	0.6782 (3)	0.65485 (12)	0.0408 (4)	
H10A	0.5007	0.6487	0.7073	0.049*	
C11	0.48936 (11)	0.8531 (3)	0.60457 (12)	0.0401 (4)	
H11A	0.4478	0.9409	0.6232	0.048*	
C12	0.51674 (9)	0.9061 (3)	0.52692 (10)	0.0301 (4)	
C13	0.57751 (13)	0.7705 (4)	0.50380 (12)	0.0497 (5)	
H13A	0.5981	0.7997	0.4517	0.060*	
C14	0.60900 (12)	0.5960 (4)	0.55329 (12)	0.0478 (5)	
H14A	0.6509	0.5086	0.5349	0.057*	
C15	0.80502 (11)	0.0062 (3)	0.64265 (10)	0.0361 (4)	
H15A	0.8023	0.1630	0.6377	0.054*	
H15B	0.7485	−0.0545	0.6217	0.054*	
H15C	0.8446	−0.0496	0.6073	0.054*	
C16	0.83244 (12)	−0.3037 (3)	0.74548 (12)	0.0384 (4)	
H16A	0.8630	−0.3487	0.8031	0.058*	
H16B	0.8590	−0.3685	0.7001	0.058*	
H16C	0.7732	−0.3505	0.7378	0.058*	
C17	0.80193 (14)	−0.1264 (3)	0.93930 (11)	0.0456 (5)	
H17A	0.8451	−0.2307	0.9301	0.068*	
H17B	0.7454	−0.1875	0.9190	0.068*	
H17C	0.8092	−0.0922	1.0016	0.068*	
C18	0.75837 (15)	0.2577 (3)	0.91871 (11)	0.0477 (5)	
H18A	0.7586	0.3850	0.8818	0.072*	
H18B	0.7825	0.2945	0.9794	0.072*	
H18C	0.7000	0.2071	0.9149	0.072*	

### (II) 4,4'-Bis[3-(2,2,6,6-tetramethylpiperidin-1-yl)prop-1-yn-1-yl]-1,1'-biphenyl Atomic displacement parameters (Å^2^)

**Table:**

	*U*^11^	*U*^22^	*U*^33^	*U*^12^	*U*^13^	*U*^23^
N1	0.0261 (6)	0.0330 (7)	0.0174 (6)	−0.0037 (5)	0.0076 (5)	0.0001 (5)
C1	0.0288 (8)	0.0347 (8)	0.0231 (7)	0.0026 (6)	0.0103 (6)	0.0023 (6)
C2	0.0262 (8)	0.0442 (10)	0.0398 (9)	0.0021 (7)	0.0102 (7)	0.0145 (8)
C3	0.0311 (9)	0.0553 (12)	0.0456 (10)	−0.0071 (8)	−0.0075 (8)	0.0224 (9)
C4	0.0568 (11)	0.0472 (11)	0.0241 (8)	−0.0167 (9)	−0.0104 (7)	0.0098 (7)
C5	0.0508 (10)	0.0364 (9)	0.0169 (7)	−0.0088 (7)	0.0088 (6)	0.0027 (6)
C6	0.0267 (8)	0.0330 (8)	0.0359 (8)	−0.0049 (6)	0.0120 (6)	−0.0015 (7)
C7	0.0241 (7)	0.0389 (9)	0.0335 (8)	−0.0034 (7)	0.0103 (6)	−0.0075 (7)
C8	0.0243 (7)	0.0387 (9)	0.0347 (8)	−0.0037 (7)	0.0092 (6)	−0.0092 (7)
C9	0.0217 (7)	0.0372 (9)	0.0325 (8)	−0.0017 (6)	0.0040 (6)	−0.0091 (7)
C10	0.0374 (9)	0.0434 (10)	0.0475 (10)	0.0033 (8)	0.0227 (8)	−0.0001 (8)
C11	0.0343 (9)	0.0417 (10)	0.0500 (10)	0.0072 (7)	0.0222 (8)	−0.0021 (8)
C12	0.0222 (7)	0.0376 (9)	0.0306 (8)	−0.0012 (6)	0.0048 (6)	−0.0111 (7)
C13	0.0514 (11)	0.0697 (14)	0.0335 (9)	0.0281 (10)	0.0221 (8)	0.0066 (9)
C14	0.0464 (11)	0.0649 (13)	0.0363 (9)	0.0264 (9)	0.0186 (8)	0.0009 (9)
C15	0.0453 (10)	0.0444 (10)	0.0203 (7)	0.0054 (8)	0.0105 (7)	−0.0009 (7)
C16	0.0436 (10)	0.0342 (9)	0.0400 (9)	0.0042 (7)	0.0148 (7)	0.0009 (7)
C17	0.0688 (13)	0.0434 (11)	0.0286 (8)	−0.0070 (9)	0.0193 (8)	0.0090 (8)
C18	0.0838 (15)	0.0393 (10)	0.0254 (8)	−0.0073 (10)	0.0239 (9)	−0.0047 (7)

### (II) 4,4'-Bis[3-(2,2,6,6-tetramethylpiperidin-1-yl)prop-1-yn-1-yl]-1,1'-biphenyl Geometric parameters (Å, º)

**Table:**

N1—C6	1.4688 (19)	C9—C10	1.392 (2)
N1—C5	1.4793 (18)	C10—C11	1.380 (3)
N1—C1	1.4804 (19)	C10—H10A	0.9500
C1—C2	1.524 (2)	C11—C12	1.403 (2)
C1—C15	1.532 (2)	C11—H11A	0.9500
C1—C16	1.542 (2)	C12—C13	1.389 (2)
C2—C3	1.516 (2)	C12—C12^i^	1.480 (3)
C2—H2A	0.9900	C13—C14	1.373 (3)
C2—H2B	0.9900	C13—H13A	0.9500
C3—C4	1.517 (3)	C14—H14A	0.9500
C3—H3A	0.9900	C15—H15A	0.9800
C3—H3B	0.9900	C15—H15B	0.9800
C4—C5	1.529 (2)	C15—H15C	0.9800
C4—H4A	0.9900	C16—H16A	0.9800
C4—H4B	0.9900	C16—H16B	0.9800
C5—C18	1.531 (3)	C16—H16C	0.9800
C5—C17	1.542 (2)	C17—H17A	0.9800
C6—C7	1.471 (2)	C17—H17B	0.9800
C6—H6A	0.9900	C17—H17C	0.9800
C6—H6B	0.9900	C18—H18A	0.9800
C7—C8	1.198 (2)	C18—H18B	0.9800
C8—C9	1.429 (2)	C18—H18C	0.9800
C9—C14	1.381 (2)		
			
C6—N1—C5	114.19 (12)	C14—C9—C8	120.16 (15)
C6—N1—C1	113.49 (12)	C10—C9—C8	122.07 (15)
C5—N1—C1	120.92 (13)	C11—C10—C9	120.43 (16)
N1—C1—C2	108.79 (13)	C11—C10—H10A	119.8
N1—C1—C15	108.16 (12)	C9—C10—H10A	119.8
C2—C1—C15	106.36 (13)	C10—C11—C12	122.43 (15)
N1—C1—C16	114.57 (13)	C10—C11—H11A	118.8
C2—C1—C16	110.15 (14)	C12—C11—H11A	118.8
C15—C1—C16	108.47 (14)	C13—C12—C11	115.55 (16)
C3—C2—C1	113.30 (13)	C13—C12—C12^i^	122.06 (18)
C3—C2—H2A	108.9	C11—C12—C12^i^	122.39 (17)
C1—C2—H2A	108.9	C14—C13—C12	122.53 (16)
C3—C2—H2B	108.9	C14—C13—H13A	118.7
C1—C2—H2B	108.9	C12—C13—H13A	118.7
H2A—C2—H2B	107.7	C13—C14—C9	121.32 (16)
C2—C3—C4	108.70 (14)	C13—C14—H14A	119.3
C2—C3—H3A	109.9	C9—C14—H14A	119.3
C4—C3—H3A	109.9	C1—C15—H15A	109.5
C2—C3—H3B	109.9	C1—C15—H15B	109.5
C4—C3—H3B	109.9	H15A—C15—H15B	109.5
H3A—C3—H3B	108.3	C1—C15—H15C	109.5
C3—C4—C5	113.55 (15)	H15A—C15—H15C	109.5
C3—C4—H4A	108.9	H15B—C15—H15C	109.5
C5—C4—H4A	108.9	C1—C16—H16A	109.5
C3—C4—H4B	108.9	C1—C16—H16B	109.5
C5—C4—H4B	108.9	H16A—C16—H16B	109.5
H4A—C4—H4B	107.7	C1—C16—H16C	109.5
N1—C5—C4	108.43 (12)	H16A—C16—H16C	109.5
N1—C5—C18	107.54 (14)	H16B—C16—H16C	109.5
C4—C5—C18	108.02 (15)	C5—C17—H17A	109.5
N1—C5—C17	114.40 (14)	C5—C17—H17B	109.5
C4—C5—C17	109.65 (15)	H17A—C17—H17B	109.5
C18—C5—C17	108.61 (14)	C5—C17—H17C	109.5
N1—C6—C7	112.04 (13)	H17A—C17—H17C	109.5
N1—C6—H6A	109.2	H17B—C17—H17C	109.5
C7—C6—H6A	109.2	C5—C18—H18A	109.5
N1—C6—H6B	109.2	C5—C18—H18B	109.5
C7—C6—H6B	109.2	H18A—C18—H18B	109.5
H6A—C6—H6B	107.9	C5—C18—H18C	109.5
C8—C7—C6	177.40 (16)	H18A—C18—H18C	109.5
C7—C8—C9	175.42 (16)	H18B—C18—H18C	109.5
C14—C9—C10	117.74 (16)		
			
C6—N1—C1—C2	170.72 (12)	C1—N1—C5—C17	−75.06 (19)
C5—N1—C1—C2	−47.93 (18)	C3—C4—C5—N1	−50.81 (19)
C6—N1—C1—C15	55.59 (17)	C3—C4—C5—C18	−167.07 (14)
C5—N1—C1—C15	−163.06 (14)	C3—C4—C5—C17	74.74 (17)
C6—N1—C1—C16	−65.52 (17)	C5—N1—C6—C7	109.77 (15)
C5—N1—C1—C16	75.83 (18)	C1—N1—C6—C7	−106.21 (15)
N1—C1—C2—C3	51.04 (19)	C14—C9—C10—C11	1.0 (3)
C15—C1—C2—C3	167.34 (15)	C8—C9—C10—C11	179.33 (16)
C16—C1—C2—C3	−75.31 (19)	C9—C10—C11—C12	−0.6 (3)
C1—C2—C3—C4	−57.6 (2)	C10—C11—C12—C13	0.0 (3)
C2—C3—C4—C5	57.58 (19)	C10—C11—C12—C12^i^	−179.94 (18)
C6—N1—C5—C4	−171.25 (14)	C11—C12—C13—C14	0.0 (3)
C1—N1—C5—C4	47.65 (19)	C12^i^—C12—C13—C14	180.0 (2)
C6—N1—C5—C18	−54.69 (17)	C12—C13—C14—C9	0.5 (3)
C1—N1—C5—C18	164.21 (14)	C10—C9—C14—C13	−0.9 (3)
C6—N1—C5—C17	66.04 (19)	C8—C9—C14—C13	−179.33 (18)

Symmetry code: (i) −*x*+1, −*y*+2, −*z*+1.
